# Comparison of the diagnostic yield of aCGH and genome-wide sequencing across different neurodevelopmental disorders

**DOI:** 10.1038/s41525-021-00188-7

**Published:** 2021-03-25

**Authors:** Francisco Martinez-Granero, Fiona Blanco-Kelly, Carolina Sanchez-Jimeno, Almudena Avila-Fernandez, Ana Arteche, Ana Bustamante-Aragones, Cristina Rodilla, Elvira Rodríguez-Pinilla, Rosa Riveiro-Alvarez, Saoud Tahsin-Swafiri, Maria Jose Trujillo-Tiebas, Carmen Ayuso, Marta Rodríguez de Alba, Isabel Lorda-Sanchez, Berta Almoguera

**Affiliations:** 1 Department of Genetics and Genomics, IIS–Fundación Jiménez Díaz University Hospital, Madrid, Spain; 2 Center for Biomedical Network Research on Rare Diseases (CIBERER), ISCIII, Madrid, Spain; 3 Department of Genetics, 12 de Octubre University Hospital, Madrid, Spain

**Keywords:** Molecular medicine, Neurodevelopmental disorders

## Abstract

Most consensus recommendations for the genetic diagnosis of neurodevelopmental disorders (NDDs) do not include the use of next generation sequencing (NGS) and are still based on chromosomal microarrays, such as comparative genomic hybridization array (aCGH). This study compares the diagnostic yield obtained by aCGH and clinical exome sequencing in NDD globally and its spectrum of disorders. To that end, 1412 patients clinically diagnosed with NDDs and studied with aCGH were classified into phenotype categories: global developmental delay/intellectual disability (GDD/ID); autism spectrum disorder (ASD); and other NDDs. These categories were further subclassified based on the most frequent accompanying signs and symptoms into isolated forms, forms with epilepsy; forms with micro/macrocephaly and syndromic forms. Two hundred and forty-five patients of the 1412 were subjected to clinical exome sequencing. Diagnostic yield of aCGH and clinical exome sequencing, expressed as the number of solved cases, was compared for each phenotype category and subcategory. Clinical exome sequencing was superior than aCGH for all cases except for isolated ASD, with no additional cases solved by NGS. Globally, clinical exome sequencing solved 20% of cases (versus 5.7% by aCGH) and the diagnostic yield was highest for all forms of GDD/ID and lowest for Other NDDs (7.1% versus 1.4% by aCGH) and ASD (6.1% versus 3% by aCGH). In the majority of cases, diagnostic yield was higher in the phenotype subcategories than in the mother category. These results suggest that NGS could be used as a first-tier test in the diagnostic algorithm of all NDDs followed by aCGH when necessary.

## Introduction

The term neurodevelopmental disorder (NDD) has been applied to a very broad group of disabilities involving the disruption of brain and neurocognitive development and includes a wide range of neurological and psychiatric problems that are clinically and causally disparate^1^.

In its latest version, the Diagnostic and Statistical Manual of Mental Disorders (DSM-5) classifies a heterogeneous group of conditions as NDD: Global developmental delay/intellectual disability (GDD/ID); Autism spectrum disorder (ASD); Attention deficit/hyperactivity disorders (ADHD); Communication disorders; Specific learning disorders; and motor disorders^2^. All these conditions have in common the impact in neuronal development, which affect various aspects of daily functioning.

NDDs are highly complex disorders characterized both by clinical and genetic heterogeneity, which makes genetic diagnosis challenging. The genetic architecture of NDDs is complex with monogenic and multifactorial inheritance^1,3,4^; with virtually any type of genetic variation involved, and over 2000 genes described to date (Deciphering Developmental Disorders, www.ddduk.org). This complexity typically requires iterative genetic testing, which frequently results in what has been called a “diagnostic odyssey”, where after a lengthy and costly process, as high as 50% of patients do not benefit of an etiological diagnosis^4,5^. Establishing a precise and timely diagnosis is critical for patient and family management and for exploring potential therapeutic interventions^6,7^

To date, only GDD/ID and ASD have recommendations for genetic testing. Current guidelines date of 2010 and include chromosomal microarray (CMA, i.e. single nucleotide polymorphism and comparative genomic hybridization—CGH—arrays) and Fragile-X testing as first-tier tests for individuals with unexplained GDD/ID and/or ASD^8–10^. The reported molecular diagnostic yield of CMA for individuals with GDD/ID, ASD, and/or multiple congenital anomalies ranges from 10 to 20% depending on the series examined^10–12^.

There is a growing body of evidence of the diagnostic superiority of next generation sequencing (NGS), whole exome sequencing (WES), and whole genome sequencing (WGS), over CMA^5^. In NDD, diagnostic rates reported in recent studies and meta-analyses are 30–40%^5,7,13–16^ and higher when trio analysis is performed^5^.

While all studies agree in the use of NGS as the first approach to NDDs^5,16^, no accepted guidelines exist yet for their systematic use in clinical practice.

For the past 5 years, the algorithm used for the diagnosis of NDDs at our Genetics Department, at Fundación Jiménez Díaz University Hospital (FJD), has followed the existing guidelines: array CGH (aCGH) and/or Fragile-X testing as the first-tier tests in patients with GDD/ID, ASD and other forms of NDD, followed by screening of ID genes by clinical exome sequencing in cases not solved by the first approach. Clinical exome sequencing refers to gene panels that typically target 4500–5000 known disease-associated genes and has demonstrated to provide a cost-effective sequencing analysis and easier interpretation of the results, avoiding or minimizing the unexpected or incidental findings, than when applying the whole exome or genome^17–19^.

**Fig. 1:**
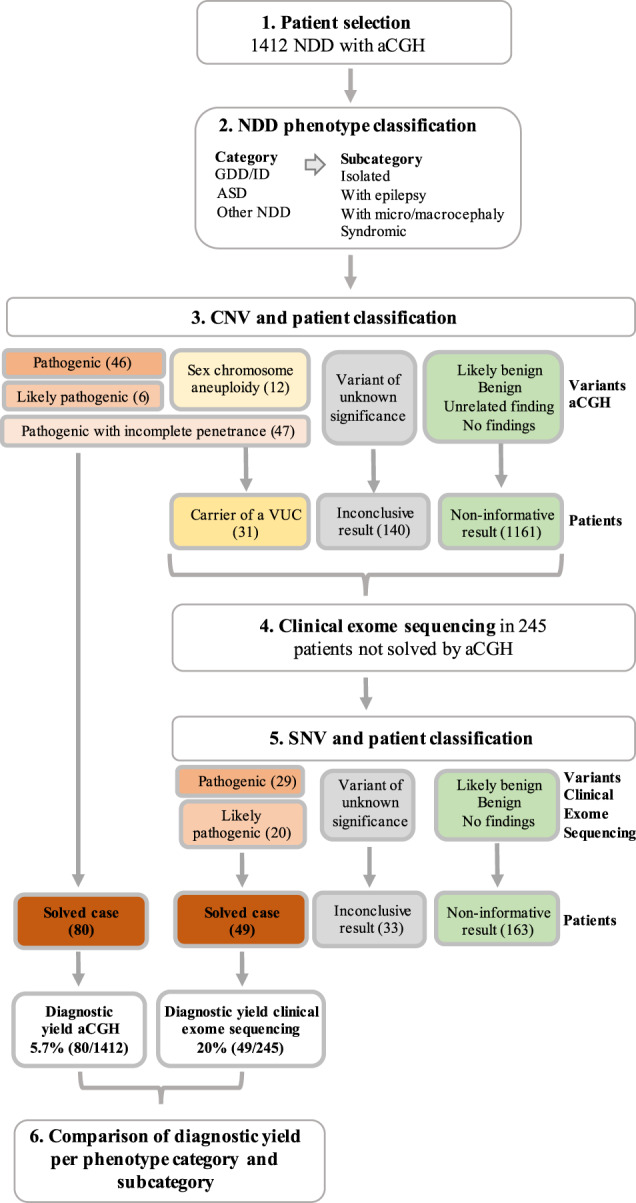
Flow chart of the process followed in this study. One thousand four hundred and twelve NDD patients with aCGH data were selected and phenotype was classified into three categories: GDD/ID, ASD, and Other NDDs; and four subcategories: isolated forms, forms with epilepsy, forms with micro or macrocephaly, and syndromic forms. CNVs identified by aCGH were classified into seven different classes according to the pathogenicity and patients carrying such variants were subsequently classified into four different categories: solved patients, carriers of a variant with unclear contribution, patients with inconclusive results and patients with non-informative results. Two hundred and forty-five unsolved patients were further subjected to clinical exome sequencing; SNVs classified into the five ACMG classes, which were translated into three different patients’ categories. Diagnostic yield, expressed as the percentage of solved cases, by aCGH and clinical exome sequencing was compared.

Using data generated by aGCH and clinical exome sequencing, we aimed at determining the diagnostic yield obtained by aCGH in NDD globally and also in its spectrum of disorders. To that end, patients were classified into different categories (NDD diagnosis) and subcategories based on the most frequent accompanying clinical signs and symptoms to minimize the heterogeneity between patients. Then, the diagnostic yield of aCGH was compared to that achieved by clinical exome sequencing for each phenotype category and subcategory. The process followed in this study is illustrated in Fig. 1.

## Results

### Characteristics of the patients included in the study and CNVs identified by aCGH

The number of patients included in the study with a diagnosis of NDD with an aCGH result was 1412: 1010 males (71.5%) and 402 females (28.5%) and the mean age of patients at the time of the aCGH testing (±SD) was 8 ± 6 years (range of 0–64years). The number of patients in each phenotype category and subcategory is illustrated in Table 1. Of the 1412 patients, 245 were subjected to clinical exome sequencing (17.3%) and 247 to Fragile-X testing (17.5%).

**Table 1:** Number and percentage of patients in each of the groups based on the aCGH results and stratified by phenotype category and subcategory.

Phenotype	Solved *N* (%)	Carrier of a VUC *N* (%)	Inconclusive *N* (%)	Unsolved *N* (%)	Total patients *N*
GDD/ID	64 (8.4%)	18 (2.3%)	80 (10.4%)	604 (78.9%)	766
GDD/ID isol	38 (6.9%)	11 (2%)	56 (10.1%)	449 (81.0%)	554
GDD/ID + epi	6 (16.7%)	3 (8.3%)	4 (11.1%)	23 (63.9%)	36
GDD/ID + micro/macro	3 (8.3%)	0 (–)	1 (2.8%)	32 (88.9%)	36
GDD/ID synd	17 (12.1%)	4 (2.9%)	19 (13.6%)	100 (71.4%)	140
ASD	13 (3.0%)	7 (1.6%)	46 (10.5%)	373 (84.9%)	439
ASD isol	11 (2.8%)	6 (1.6%)	39 (10.1%)	330 (85.5%)	386
ASD + epi	0 (–)	0 (–)	2 (16.7%)	10 (83.3%)	12
ASD + micro/macro	0 (–)	0 (–)	2 (14.3%)	12 (85.7%)	14
ASD synd	2 (7.4%)	1 (3.7%)	3 (11.1%)	21 (77.8%)	27
Other NDDs	3 (1.4%)	6 (2.9%)	14 (6.8%)	184 (88.9%)	207
Other isol	3 (1.8%)	5 (2.9%)	11 (6.4%)	152 (88.9%)	171
Other + epi	0 (–)	0 (–)	1 (11.1%)	8 (88.9%)	9
Other + micro/macro	0 (–)	0 (–)	1 (12.5%)	7 (87.5%)	8
Other synd	0 (–)	1 (5.3%)	1 (5.3%)	17 (89.4%)	19
Total	80 (5.7%)	31 (2.2%)	140 (9.9%)	1161 (82.2%)	1412

Percentage in each group is calculated from the totals. For the phenotype: *GDD/ID* Global developmental delay/intellectual disability, *ASD* Autism spectrum disorder, *Other NDDs* Other neurodevelopmental disorders, *isol* isolated forms, *epi* epilepsy, *micro/macro* micro/macrocephaly, *synd* syndromic, *VUC* variant of unclear contribution.

In our cohort of 1412 patients, we reported findings back from the aCGH to 329 (23.3% of patients), of whom 78 were heterozygous carriers of pathogenic CNVs in recessive genes unrelated to the clinical suspicion. Of the remaining 251 patients, 52 carried a pathogenic or likely pathogenic variant, including five chromosomal rearrangements and 47 CNVs; 47 carried a variant with incomplete penetrance; 13 patients had a sex chromosome aneuploidy (1 Turner syndrome; 1 triple X syndrome; 5 Klinefelter syndrome, and 6 XYY); and 140 carried a VUS.

### Solved patients and patients with a variant of unclear contribution (VUC) by aCGH

Patients were classified into four categories based on the CNV found: (i) solved patients; (ii) carriers of a variant of unclear contribution (VUC); (iii) patients with inconclusive results; and (iv) unsolved patients. Results are illustrated in Table 1 and Fig. 1.

Of the 52 carriers of a pathogenic/likely pathogenic variant, there were three for whom causality could not be definitively established due to being females with a X-linked CNV inherited from an unaffected mother or with no information about the inheritance (families RM-0515, RM-0888, and RM-1619). Proband from family RM-0515 carried a heterozygous 6 Mb deletion at Xq28 (chrX:149247622-155221913) that was also shared with her unaffected sister. X-chromosome inactivation (XCI) testing in the three females of this family demonstrated a completely skewed pattern with the X chromosome carrying the deletion, being the inactive in all three females. Further clinical exome sequencing identified a de novo pathogenic variant in *GRIN2B* (MIM *138252) in the proband, which completely explained the phenotype, i.e. intellectual disability with a psychiatric disorder (MIM #613970). The proband female from family RM-0888, who had GDD at the time of referral, carried a maternally inherited partial duplication of *IL1RAPL1* (chrX:29251326-29670652), a gene associated with X-linked ID (MIM #300143), which included the coding region. Results for XCI are still pending due to the current unavailability of DNA samples. The third female (RM-1619) carried a de novo deletion at Xq28 (chrX:152925630-153027220), spanning the genes *ABCD1*, associated with X-linked adrenoleukodystrophy (MIM #300100), *BCAP31*, associated with X-linked deafness, dystonia, and cerebral hypomyelination (MIM #300475), *SLC6A8*, associated with cerebral creatine deficiency syndrome (MIM #300352), and *PNCK* (MIM *300680). This patient had GDD, atypical febrile seizures, microcephaly, and short stature. XCI results were non-informative due to homozygosity of the HUMARA locus and further clinical exome sequencing failed to identify another pathogenic variant in this patient. The three cases were reclassified as carriers of a VUC by aCGH.

Carriers of variants with incomplete penetrance (*n* = 47) were further classified into either solved patients or carriers of a VUC. All patients with a sex chromosome aneuploidy were classified as patients with a VUC: eight with GDD/ID, two with ASD, and two with Other NDDs.

Also, carriers of the 15q11.2BP1-BP2 deletion (*n* = 8) and patients with a variant of incomplete penetrance that were subjected to additional genetic testing after the aCGH (*n* = 7) were considered carriers of a VUC.

Finally, 80 patients were classified as solved cases (Fig. 1) (49 with pathogenic/likely pathogenic variants—Table 2—and 31 with a variant of incomplete penetrance—Table 3—) (5.7%) and 31 as carriers of a VUC (12 with a sex chromosome aneuploidy, 16 with a variant of incomplete penetrance and the three females with the variants in the X chromosome) (2.2%).

**Table 2:** Pathogenic and likely pathogenic variants identified in the solved patients. For the genetic diagnosis, MIM numbers are provided when available.

Genetic diagnosis (MIM #)	Genomic coordinates (CNV type)	Size	Class	Rationale for the classification	Phenotype (gender)	Inher
1p36.33-p36.32 duplication	chr1:835601-3648438	2.8–3.6	P	ClinVar and DECIPHER with similar CNV classified as P/LP	GDD/ID + epi (F)	Inh
1p21.3-p13.3 deletion	chr1:98429416-111608309	13.2–13.3	P	Known variant/syndrome	GDD/ID + epi (M)	NA
2q13 deletion	chr2:109737782-114719444	1.7–2.2	P	Known variant/syndrome	GDD/ID_isol (M)	DN
2q22.3-q23.3 duplication	chr2:144709544-154452355	9.7–10	P	Known variant/syndrome	GDD/ID_synd (M)	DN
3q26.32 deletion	chr3:176720110-177692976	0.97–1.2	P	*TBL1XR1* known GDD gene	GDD/ID_isol (M)	DN
3q29 deletion syndrome	chr3:195769570-197332976	1.6–1.8	P	Known variant/syndrome	GDD/ID + micro/macro (M)	DN
4q13.2-q13.3 deletion	chr4:68155831-73330955	5.2–5.4	P	ClinVar and literature with similar CNV classified as P/LP	GDD/ID_synd (F)	NA
7q22.1 deletion	chr7:101668049-103143668	1.5–1.6	P	*RELN* known GDD/epilepsy gene	GDD/ID + epi (F)	NA
AUTS2 syndrome (MIM #615834)	chr7:67767963-69320956 (dup)	1.6–1.7	P	Known variant/syndrome	GDD/ID_isol (M)	DN
	chr7:69564262-695927 (del)	0.03–0.06	P		ASD_isol (M)	DN
7q11.23 duplication syndrome	chr7:72221649-74339044	2.1–3.2	P	Known variant/syndrome	GDD/ID_isol (F)	NA
	chr7:72745047-74138460	1.4–2.1	P		GDD/ID_synd (F)	DN
10q11.22-q11.23 deletion	chr10:49392896-52362158	3.0–4.0	LP	ClinVar and DECIPHER with similar CNV classified as P/LP	GDD/ID_isol (M)	NA
11p15.5 deletion	chr11:205827-697426	0.49–0.74	P	Possible candidate genes: *DEAF1, DRD4*	ASD_isol (F)	DN
Lamb–Shaffer syndrome (MIM #616803)	chr12:23916595-24134081 (del)	0.22–0.25	P	Known variant/syndrome	GDD/ID_synd (F)	DN
12q21.2-q21.31 duplication	chr12:79831049-85693154	5.9–6.03	LP	ClinVar and DECIPHER with similar CNV classified as P/LP	GDD/ID_isol (M)	NA
*FGF14* deletion (MIM #609307)	chr13:102992332-103117739	0.13–0.15	LP	Known gene	GDD/ID_isol (F)	DN
15q11-q13 duplication syndrome	chr15:22822019-28513165 (x3 individuals)	5.7–29	P	Known variant/syndrome	GDD/ID_isol (2F, 1M)	DN
(MIM #608636)	chr15:22822019-32438943	5.7–29	P	Known variant/syndrome	GDD/ID_isol (F)	DN
Angelman syndrome (MIM #105830)	chr15:23717628-28513165 (del)	4.8–6.1	P	Known variant/syndrome	GDD/ID_synd (M)	NA
Prader–Willi syndrome (MIM #176270)	chr15:23717628-28513165 (del)	4.8–6.1	P	Known variant/syndrome	Other_isol (M)	NA
15q26 microdeletion	chr15:94995432-993443687	4.4–4.5	P	Known variant/syndrome	GDD/ID_synd (F)	DN
Smith Magenis syndrome (MIM #182290)	chr17:16814853-20079020 (del)	0.50–0.53	P	Known variant/syndrome	GDD/ID_isol (M)	NA
17q12 recurrent deletion syndrome (MIM #614527)	chr17:34816424-36207539	1.4–2.0	P	Known variant/syndrome	GDD/ID_isol (F)	NA
Koolen de Vries syndrome (MIM #610443)	chr17:43713616-44275738 (del)	0.6–1.30	P	Known variant/syndrome	GDD/ID_synd (M)	NA
17p13.3 duplication syndrome (MIM #613215)	chr17:78346-347175	3.4–3.5	P	Known variant/syndrome	GDD/ID_synd (F)	DN
18q12.1-q12.3 deletion	chr18:32241257-38294860	6.1–6.2	P	Known variant/syndrome (*CELF4* candidate gene)	GDD/ID_isol (F)	DN
18q- syndrome (MIM #601808)	chr18:68240572-76669641 (del)	8.4–8.6	P	Known variant/syndrome	GDD/ID_synd (M)	NA
	chr18:72343121-78013620 (del)	5.7–5.8		Known variant/syndrome	GDD/ID_synd (M)	DN
22q11.21 duplication	chr22:20734765-21417548	0.68–1.6	P	Known variant/syndrome	ASD_isol (M)	DN
22q11.2 duplication syndrome	chr22:18919528-21349219	2.5–3.2	P	Known variant/syndrome	GDD/ID_isol (F)	DN
22q11.2 deletion syndrome (MIM #18840)	chr22:18919528-21417548	2.5–3.2	P	Known variant/syndrome	GDD/ID_isol (F); GDD/ID_synd (M); Other_isol (M)	DN
22q11.2 microdeletion (MIM #611867)	chr22:21798705-22951375	1.2–1.7	P	Known variant/syndrome	GDD/ID_synd (F)	NA
Phelan McDermid syndrome (MIM #606232)	chr22:51137326-51178150 (del)	0.04–0.17	P	Known variant/syndrome	GDD/ID + epi (F); GDD/ID_isol (M)	NA; DN
Xq28 duplication syndrome (MIM #300815)	chrx:153578515-153783185	0.20–0.26	P	Known variant/syndrome	GDD/ID + micro/macro (M)	Inh
Xq25 microduplication syndrome	chrX:123002811-123237762	0.24–0.34	P	Known variant/syndrome	GDD/ID_isol (M)	NA
Partial duplication of *IL1RAPL1* (MIM #300143)	chrX:29595176-30070288	0.48–0.52	LP	Known gene and mechanism	GDD/ID_synd (M)	NA
Xp22.32-22.2 deletion (MIM #308100, #308700, #300000)	chrX:5748782-10477366	4.7–4.8	P	Known variant/syndrome	GDD/ID_synd (F)	DN
ATRX syndrome (MIM #301040)	chrX:76411307-76972336 (del)	0.56–0.71	P	Known variant/syndrome	ASD_isol (M)	DN
Unbalanced product of parental translocations/Chromosomal rearrangement	der(12)t(7;12)(p22.3p13.33)	–	P		ASD_isol (F)	–
	der(18)t(9;18)(p23;p11.31)	–	P		GDD/ID_isol (F)	–
	Rearrangement chr18	–	P		GDD/ID_isol (M)	–
	Complex rearrangement chr9	–	P		GDD/ID_isol (F)	–
	Complex rearrangement chr1	–	P		GDD/ID_synd (M)	–

Genomic coordinates correspond to hg19. For the type of CNV *del* deletion, *dup* duplication. Size includes the minimum and maximum estimated size in Mb; *NA* not available; For the inheritance, *DN* de novo, *Inh* inherited, *NA* not available. For the variant class, *LP* likely pathogenic and *P* pathogenic. For the phenotype: *ASD* Autism spectrum disorder, *GDD/ID* Global developmental delay/intellectual disability, *Other NDDs* Other neurodevelopmental disorders, *epi* epilepsy, *isol* isolated forms *micro/macro* micro/macrocephaly, *synd* syndromic, *F* female, *M* male.

**Table 3:** Pathogenic variants with incomplete penetrance identified in the solved patients.

Genetic diagnosis (MIM #)	Genomic coordinates	Size	Phenotype (gender)	Inheritance
1q21.1 BP2-BP3 duplication (MIM #612475)	chr1:145400040-145746492	0.34–1.2	GDD/ID_isol (F); GDD/ID + micro/macro (F)	NA
	chr1:145400040-146531598	0.34–1.2	ASD_isol(M)	Inh
2p16.3 (*NRXN1*) deletion (MIM #614332)	chr2:50557535- 50766750	0.21–0.23	ASD_isol (M)	Inh
	chr2:48503210-51396916	0.29–0.30	GDD/ID_isol (F)	DN
15q13.3 microdeletion (MIM #612001)	chr15:31140606-32438944	1.3–2.8	GDD/ID_isol (M)	Inh
	chr15:31140606-32438944	1.3–2.8	GDD/ID_isol (M)	DN
	chr15:29214105-32438943	1.3–2.8	GDD/ID_isol (F)	Inh
	chr15:31140606-32438943	1.3–2.8	GDD/ID_synd (M)	Inh
	chr15:31140606-32438944	1.3–2.8	GDD/ID + epi (M)	Inh
	chr15:31140606-32438944	1.3–2.8	GDD/ID_isol (M)	Inh
15q13.3 microduplication	chr15:32029550-32438944	0.4–1.0	ASD_isol (M)	Inh
	chr15:32029550-32438943	0.4–1.0	ASD_synd (F)	Inh
	chr15:32029550-32438943	0.4–1.0	GDD/ID_isol (F)	Inh
	chr15:31140606-32438944	0.4–1.0	Other_isol (F)	Inh
16p13.11 microdeletion	chr16:15049829-16287899	1.2–2.1	GDD/ID_isol (F)	Inh
16p13.11 microduplication	chr16:15049829-16287899	1.2–2.1	GDD/ID_isol (F)	NA
	chr16:15125829-16287899	1.2–2.1	GDD/ID_isol (M)	NA
	chr16:15049829-16287899	1.2–2.1	GDD/ID_isol (M)	Inh
16p13.11 deletion	chr16:15512480-18128489	2.6–3.4	GDD/ID_isol (M)	Inh
16p12.2 recurrent deletion	chr16:21950360-22428364	0.5–0.9	ASD_isol (M)	Inh
16p11.2 microdeletion (MIM #611913)	chr16:28488583-30188269	0.5–1.3	ASD_isol (F)	DN
	chr16:28488583-30188269	0.5–1.3	GDD/ID + epi (F)	DN
	chr16:28488583-30188269	0.5–1.3	GDD/ID_isol (M)	NA
	chr16:29664618-30171078	0.5–1.2	GDD/ID_synd (M)	DN
	chr16:29664618-30171078	0.5–1.2	GDD/ID_isol (F)	DN
16p11.2 BP2-BP3 deletion (MIM #613444)	chr16:28833437-29038072	0.2–1.6	ASD_synd (F)	Inh
	chr16:28833437-29038072	0.2–1.6	GDD/ID_isol (M)	Inh
16p11.2 microduplication (MIM #614671)	chr16:29657192-30188268	0.5–1.3	GDD/ID_isol (M)	Inh
17q12 recurrent duplication (MIM #614526)	chr17:34816424-36207539	1.4-2.0	GDD/ID_isol (M)	NA
22q11.2 microduplication (MIM #608363)	chr22:18919528-21417548	2.5–3.2	ASD_isol (M)	NA

For the genetic diagnosis, MIM numbers are provided when available. Genomic coordinates correspond to hg19. For the type of CNV *del* deletion, *dup* duplication. Size includes the minimum and maximum estimated size in Mb; *NA* not available; For the inheritance, *DN* de novo, *Inh* inherited, *NA* not available. For the phenotype: *ASD* Autism spectrum disorder, *GDD/ID* Global developmental delay/intellectual disability, *Other NDDs* Other neurodevelopmental disorders; *epi* epilepsy, *isol* isolated forms *micro/macro* micro/macrocephaly, *synd* syndromic, *F* female, *M* male.

### Patients with inconclusive and non-informative results by aCGH

One hundred and forty patients were classified as cases with inconclusive results due to carrying a VUS (9.9%; Table 1; Fig. 1). There were some recurrent VUS: CNVs involving different genes from the contactin protein family (*CNTNAP2* -7q35-, *CNTNAP4* -16q23.1-, *CNTN4* -3p26.3-, and *CNTN6* -3p26.3-) in nine patients; the 15q11.2 BP1-BP2 microduplication in nine patients; CNVs involving the gene *RBFOX1* (16p13.3) in eight patients; the partial duplication of *DISC1* (1q42.2) in six patients and CNVs involving *DPP6* (7q36.2) in five patients.

One thousand one hundred and sixty-one patients were classified as unsolved patients with a non-informative aCGH result (Fig. 1).

### Diagnostic yield and comparison of aCGH and clinical exome sequencing in the different phenotype categories and subcategories

Clinical exome sequencing was performed in 245 patients of the 1332 patients not solved by aCGH (carriers of VUC, patients with inconclusive and non-informative results; 18.4%). The proportion of patients sequenced across the phenotype categories was 23.9% of GDD/ID (168 of 702 patients not solved), 11.5% of ASD (49 of 426 patients not solved), and 13.7% of Other NDDs (28 of 204 patients not solved). Across the different phenotype subcategories, the groups with the highest proportion of patients sequenced were forms of NDD with epilepsy (50%, 33.3%, and 55.6%, for GDD/ID, ASD, and Other NDDs, respectively) and syndromic forms (61.7%, 36%, and 31.6%, for GDD/ID, ASD and Other NDDs, respectively); followed by forms with micro/macrocephaly (27.3%, 28.6%, and 25%, for GDD/ID, ASD, and Other NDDs, respectively). Isolated forms were the least represented in the sequenced group) with 13.2%, 8.3%, and 8.9%, for GDD/ID, ASD, and Other NDDs, respectively.

Forty-five patients were sequenced using the True Sight One (TSO) and 200 with the Clinical Exome Solution (CES) and the virtual panels used included 1349 and 1369 genes, respectively (DDD panel). Results found were 49 patients solved (29 with pathogenic and 20 with likely pathogenic variants; 20%; Table 4); 33 patients with inconclusive results, due to carrying a VUS (13.5%) and 163 unsolved patients with non-informative results (66.5%). No significant differences were found in the results obtained by TSO and CES (data not shown). All patients sequenced using CES were subjected to the CNV analysis pipeline implemented in the DDM platform, but no additional CNVs were identified. There were 29 samples with 31 variants identified by aCGH that were subsequently identified with the CNV analysis algorithm: one pathogenic variant (the 6 Mb Xq28 deletion) two sex chromosome aneuploidy (XYY); three variants with incomplete penetrance (15q11.2 BP1-BP2 deletion; 15q13.3 microduplication and a 2p16.3 deletion involving *NRXN1*) and 25 VUS (see Supplementary Table 1). Eighteen of the 31 variants were detected by the CES CNV analysis (58.1%), including the two XYY cases, the pathogenic CNV and the three variants with incomplete penetrance. Genomic coordinates of the CNVs by CES are detailed in Supplementary Table 1. Of the 13 variants that were not detected by CES, 11 CNVs fell in regions not covered by CES and only two had genes targeted by the clinical exome. Of the CNVs potentially identifiable by CES (*n* = 20), 90% were accurately detected.

**Table 4:** Genetic diagnosis of the 49 patients solved by clinical exome sequencing.

Genetic diagnosis (MIM #)—gene (transcript)	Variant (cDNA and protein)	Inheritance	Class	Phenotype (gender)
KBG syndrome AD (MIM #148050)- *ANKRD11* (NM_001256182)	c.3193 A > T, p.Lys1065*	NA	LP	GDD/ID_synd (M)
	c.548_551delGCAT, p.Arg183Profs*44	NA	LP	GDD/ID_synd (M)
	c.2765_2766del, p.Glu922Alafs*6 (x2)	DN (x2)	P	GDD/ID_synd (M); GDD/ID + epi (F)
	c.1903_1907delAAACA, p.Lys635Glnfs*26	NA	LP	GDD/ID_synd (M)
Mental retardation, AD 26 (MIM #615834)- *AUTS2* (NM_001127231)	c.1298del, p.Leu433Profs*40	DN	P	GDD/ID + micro/macro (M)
Noonan syndrome 7 AD (MIM #613706)- *BRAF* (NM_004333)	c.1738A > G, p.Asn580Asp	DN	P	GDD/ID_synd (F)
Intellectual developmental disorder, XL, syndrome, Snijders Blok type (MIM #300958)- *DDX3X* (NM_001193416)	c.508dupG, p.Ala170Glyfs*11	DN	P	GDD/ID_isol (F)
Mental retardation, XL 90 (MIM #300850)- *DLG3* (NM_021120)	c.2266 C > T, p.Arg756*	Mat	P	GDD/ID_isol (M)
Kleefstra syndrome 1 AD (MIM #610253)- *EHMT1* (NM_024757)	c.2426 C > T, p.Pro809Leu (x2)	DN, NA	P	GDD/ID_synd (M) x2
Rubinstein-Taybi syndrome 2 AD (MIM #613684)- *EP300* (NM_001429)	c.1365delT, p.Val456Leufs*9	DN	P	GDD/ID_synd (M)
	c.4179delA, p.Tyr1394Thrfs*16	DN	P	GDD/ID_synd (F)
Intellectual developmental disorder, AD (MIM #618089)- *FBXO11* (NM_001190274)	c.1480 C > T, p.Arg494*	NA	LP	GDD/ID_synd (M)
Otopalatodigital syndrome type 1 XL (OPD1; MIM# 311300)- *FLNA* (NM_001110556)	c.586 C > T, p.Arg196Trp	Mat	LP	ASD_synd (M)
Rett syndrome, congenital variant AD (MIM #613454)- *FOXG1* (NM_005249)	c.301 C > T, p.Gln101*	DN	P	GDD/ID + micro/macro (F)
	c.553 A > T, p.Ser185Cys	DN	P	GDD/ID_isol (M)
Mental retardation, language impairment with/without autistic features, AD (MIM #613670)- *FOXP1* (NM_001244810)	c.454delG, p.Glu152Asnfs*54	NA	LP	GDD/ID_synd (M)
Epileptic encephalopathy, early infantile, 19, AD (MIM #615744)- *GABRA1* (NM_000806)	c.268 G > C, p.Asp90His	DN	P	GDD/ID + epi (M)
Polydactily pre and postaxial, AD (MIM #174700 and #174200)- GLI3* (NM_000168)	c.3640 C > T, p.Gln1214*	NA	LP	GDD/ID_isol (M)
Simpson-Golabi-Behmel syndrome, type 1, AD (MIM #312870)- *GPC3* (NM_001164617)	c.1228 C > T, p.Arg410*	DN	P	GDD/ID_synd (F)
Mental retardation, AD 6 (MIM #613970)- *GRIN2B* (NM_000834)	c.1451 G > A, p.Gly484Asp	DN	P	ASD + epi (F)
	c.2406_2414del, p.His802_Lys805delinsGln	DN	LP	GDD/ID_synd (F)
	c.1562_1563dup, p.Val522Argfs*10	NA	P	GDD/ID_isol (F)
Cornelia de Lange syndrome 5, X-linked (MIM #300882)- *HDAC8* (NM_018486)	c.808 C > T, p.Arg361*	DN	P	GDD/ID_synd (F)
Mental retardation, XL 1/78 (MIM #309530)- *IQSEC2* (NM_001111125)	c.2665_2667delATC, p.Ile889del	DN	LP	GDD/ID_synd (F)
Arboleda-Tham syndrome AD (MIM #616268)- *KAT6A* (NM_006766)	c.4120 G > T, p.Glu1374*	NA	LP	GDD/ID_synd (F)
Liang-Wang syndrome AD (MIM #618729)- *KCNMA1* (NM_002247)	c.2984 A > G, p.Asn995Ser	DN	P	GDD/ID_synd (F)
Kabuki syndrome 1, AD (MIM #147920)- *KMT2D* (NM_003482)	c.5269 C > T, p.Arg1757*	DN	P	GDD/ID_synd (M)
Poretti-Boltshauser syndrome AR (MIM #615960)- *LAMA1*(NM_005559)	c.6333dupA, p.Gln2112Thrfs*6; c.2893 G > A, p.Asp965Asn	I-Trans	P/LP	GDD/ID_synd (M)
Rett syndrome XL (MIM #312750)- *MECP2* (NM_004992)	c.1163_1188del, p.Pro388Argfs*8	DN	P	GDD/ID_isol (F)
	c.808 C > T, p.Arg270*	DN	P	GDD/ID_isol (F)
	c.378-2 A > G	DN	P	GDD/ID_isol (F)
	Deletion of ex 3 and 4^a^	DN	P	GDD/ID_isol (F)
Mental retardation, XL 98 (MIM #300912)- *NEXMIF* (NM_001008537)	c.3458dupA, p.Asn1153Lysfs*8	NA	LP	GDD/ID_synd (F)
Sotos syndrome 2, AD (MIM #614753)- *NFIX* (NM_001271043)	c.52-16 G > A	DN	LP	GDD/ID_synd (M)
Sotos syndrome 1, AD (MIM #117550)- *NSD1* (NM_022455)	c.5691 T > G, p.Cys1897Trp	NA	LP	GDD/ID_synd (M)
Niemann-Pick disease, type C1, D, AR (MIM #257220)- *NPC1* (NM_000271)	c.352_353delAG, p.Gln119Valfs*8/c.2780 C > T, p.Ala927Val	I-Trans	P	Other NDD (F)
Epileptic encephalopathy, early infantile, 9, XL (MIM #300088)- *PCDH19* (NM_001105243)	c.361 G > A, p.Asp121Asn	DN	P	GDD/ID + epi (F)
White-Sutton syndrome, AD (MIM #616364)- *POGZ* (NM_015100)	c.3118 G > A, p.Glu1040Lys	NA	LP	GDD/ID_isol (F)
Jansen de Vries syndrome, AD (MIM #617450)- *PPM1D* (NM_003620)	c.1458_1464delACATGAT, p.His487Leufs*2	DN	P	Other NDD (M)
Macrocephaly/Autism syndrome AD (MIM #605309)- *PTEN* (NM_000314)	c.633 C > G, p.Cys211Trp	NA	LP	ASD + micro/macro (M)
Glass syndrome AD (MIM #612313)- *SATB2* (NM_001172509)	c.1299 C > G, p.Tyr433*	NA	LP	GDD/ID_synd (F)
Epilepsy, generalized, with febrile seizures plus, type 2, AD (MIM #604403)- *SCN1A* (NM_001165963)	c.2672 G > A, p.Gly891Glu	S	LP	GDD/ID + epi (M)
Nicolaides-Baraitser syndrome AD (MIM #601358)- *SMARCA2* (NM_003070.5)	c.1477_1479del, p.Lys493del	DN	LP	GDD/ID_synd (LP)
Lamb-Shaffer syndrome AD (MIM #616803)- *SOX5* (NM_001261414)	c.1310 G > A; p.Arg437His	DN	LP	GDD/ID_isol (F)
Pitt-Hopkins syndrome AD (MIM #610954)- *TCF4* (NM_001083962)	c.990 G > A, p.Ser330Ser	DN	P	GDD/ID + micro/macro (M)
Mental retardation AR 13 (MIM #613192)- *TRAPPC9*(NM_031466.6)	c.3103 C > T, p.Arg1035* (homozygous)	I-Trans	LP	GDD/ID + micro/macro (F)
Mental retardation, AD 22 (MIM #612337)- *ZBTB18* (NM_205768.2)	c.1384_1385delinsTA, p.Leu462*	DN	P	GDD/ID + micro/macro (M)

For the genetic diagnosis, MIM numbers are provided; *AD* autosomal dominant, *AR* autosomal recessive, *XL* X-linked. For the inheritance, *DN* de novo, *I-Trans* inherited in trans, *NA* not available. For the variant class, *LP* likely pathogenic and *P* pathogenic. For the phenotype: *ASD* Autism spectrum disorder, *GDD/ID* Global developmental delay/intellectual disability, *Other NDDs* Other neurodevelopmental disorders, *epi* epilepsy, *isol* isolated forms, *micro/macro* micro/macrocephaly, *synd* syndromic, *F* female, *M* male. *x2* 2 patients.

^a^For the MECP2 exons 3 and 4 deletion the estimated genomic coordinates are chrX:(153248310-153295808)-(153298018-153357594).

Diagnostic yield for aCGH and clinical exome sequencing was calculated considering only patients solved (Table 5). For aCGH, overall diagnostic yield was 5.7%, with GDD/ID being the category with the highest diagnostic rate, 8.4% of solved cases. For the rest of the phenotype categories, diagnostic rates were all below 5%.

**Table 5:** Diagnostic yield for aCGH and clinical exome sequencing and statistical comparison between both tests.

	CGH array		Clinical exome sequencing		
Phenotype category and subcategory	Solved *N* (%)	Total *N*	Solved *N* (%)	Total *N*	Adjusted *p* value (Mixed effects model)
GDD/ID (all)	64 (8.4%)	766	44 (26.2%)^a^	168	4.2 × 10^−9^
GDD/ID isol	38 (6.9%)	554	11 (16.2%)^a^	68	0.029
GDD/ID + epi	6 (16.7%)	36	4 (26.7%)	15	0.740
GDD/ID + micro/macro	3 (8.3%)	36	5 (55.6%)^a^	9	0.015
GDD/ID synd	17 (12.1%)	140	24 (31.5%)^a^	76	0.004
ASD (all)	13 (3.0%)	439	3 (6.1%)	49	0.498
ASD isol	11 (2.8%)	386	0 (–)	32	1
ASD + epi	0 (–)	12	1 (25.0%)	4	1
ASD + micro/macro	0 (–)	14	1 (25.0%)	4	1
ASD synd	2 (7.4%)	27	1 (11.1%)	9	1
Other NDDs (all)	3 (1.4%)	207	2 (7.1%)	28	0.177
Other isol	3 (1.8%)	171	2 (13.3%)	15	0.065
Other + epi	0 (–)	9	0 (–)	5	1
Other + micro/macro	0 (–)	8	0 (–)	2	1
Other synd	0 (–)	19	0 (–)	6	1
Total	80 (5.7%)	1412	49 (20%)^a^	245	7.0 × 10^−12^

For the phenotype: *ASD* Autism spectrum disorder, *GDD/ID* Global developmental delay/intellectual disability, *Other NDDs* other neurodevelopmental disorders, *epi* epilepsy, *isol* isolated forms, *micro/macro* micro/macrocephaly, *synd* syndromic, *F* female, *M* male.

^a^Refers to phenotypes where the diagnostic yield of clinical exome sequencing is statistically significant compared to aCGH.

For clinical exome sequencing, overall diagnostic yield was 20%, being higher for GDD/ID (26.2%) and lower for Other NDDs (7.1%) and ASD (6.1%).

As evidenced in Table 5, for both aCGH and clinical exome sequencing, diagnostic rates were, in general, higher in the phenotype subcategories than in their respective mother category.

When both methods were compared, diagnostic rates by clinical exome sequencing were higher than those of aCGH for all phenotype categories and subcategories, except for isolated ASD. Clinical exome sequencing was significantly superior than aCGH overall (*p* = 7.1 × 10^−10^), for the diagnosis of GDD/ID (*p* = 2.4 × 10^−7^), but not for ASD or Other NDDs (*p* > 0.05).

Sex distribution of solved patients was comparable between the two methods (Fisher test *p* value > 0.05 for all categories and subcategories; data not shown).

## Discussion

Current recommendations for the genetic diagnosis of NDDs date of 2010 and include the use of a CMA, such as aCGH, as the first-tier test for individuals with unexplained GDD/ID and/or ASD^8–10^. These guidelines have not been updated since then and, over the past decade, a growing body of evidence has proven the higher resolution of NGS in the diagnosis of such conditions and supports its use as the preferred first approach^5,7,13–16^. In this regard, Srivastava et al. recently published the results of a meta-analysis of the diagnostic yield of WES reported in NDDs and concluded that this technique is superior than CMA and should be used as the first-tier test^5^.

In order to provide additional evidence for the use of NGS as the preferred test in NDDs, in the present work, we used genomic data generated on aCGH and clinical exome sequencing between 2015 and 2019, as part of our genetic diagnostic algorithm, to delineate the diagnostic yield of each technology in NDDs, and across its spectrum of disorders, and compare the performance of both. Strengths of this study are the large cohort included and that all patients were assessed by a clinical geneticist and extensive clinical and phenotypic data were available, which were used for the stratification of the main NDD phenotypes (GDD/ID, ASD, and Other NDDs) into four different phenotypic subcategories based on the most commonly observed signs and symptoms in the clinic. This stratification minimizes the clinical heterogeneity between groups and provides the diagnostic yield of aCGH and clinical exome sequencing in more homogenous NDD phenotypes that can be used in clinical practice. The a priori knowledge of the expected diagnostic rate for a specific form of NDD is critical for clinicians, patients, and parents and has a major impact on the time to diagnosis, the burden of tests to be performed and the associated costs.

As expected, we found clinical exome sequencing superior than aCGH in the genetic diagnosis of NDDs overall and also across all phenotype categories and subcategories. An exception was the subcategory isolated ASD, where no additional patient was solved by NGS. Statistical significance was achieved for GDD/ID (26.2% of solved cases by NGS versus 8.4% by aCGH). Across the different GDD/ID subcategories, NGS achieved the highest yield, 55.6%, for forms of GDD/ID with micro or macrocephaly (versus 8.3% by aCGH), 31.5% for syndromic forms of GDD/ID (versus 12.1% by aCGH), 26.7% for forms of GDD/ID with epilepsy (versus 16.7% by CGH) and 17.7% for isolated forms (versus 6.9% by aCGH). These rates are similar or even higher than those previously reported^5,7,13–16^. One factor that has to be accounted for in the diagnostic yield obtained for clinical exome sequencing is the bias in the sample selection, as all samples included were previously screened by aCGH for CNVs and large chromosomal aberrations and what may have led to an overestimation of the real resolution of clinical exome sequencing. However, the large differences found in the diagnostic rates between both methods and the current possibility of analyzing CNVs using NGS data suggest that the overestimation might have been minimal.

For ASD and Other NDDs no significant differences were found between both tests despite NGS still being superior than aCGH: 6.1% versus 3%, respectively, for ASD and 7.1% versus 1.4%, respectively, for Other NDDs. The limited sample size of patients solved in these categories may have influenced the lack of statistical significance.

The number of patients not solved by aCGH subjected to clinical exome sequencing was lower for ASD and Other NDDs than for the rest of the phenotypes: 11.5% and 13.7%, respectively, versus 23.9% of GDD/ID. When considering phenotype subcategories, the proportion of patients sequenced was higher for forms of NDD with epilepsy and syndromic forms, followed by forms of NDD with micro or macrocephaly and with isolated forms being the least represented. Criteria for performing NGS to patients with inconclusive or uninformative aCGH results in our diagnostic algorithm included a confirmed NDD diagnosis upon post-aCGH evaluation; the complexity of the phenotype; the possibility of follow-up; the availability of clinical exome sequencing at the time of the study, implemented in our laboratory at the end of 2016, and the probability of a monogenic origin of the condition in each particular patient, with most forms of ASD, and all the conditions included in the category Other NDDs, having a multifactorial inheritance^1,4^; and the existing evidence of the genetic contributors to the phenotypes at the time of testing. These selection criteria explain the relatively limited number of samples subjected to NGS in this study with a bias towards more severe, complex and monogenic disorders, might have led to overestimate the diagnostic yield of NGS.

The diagnostic yield observed for both ASD and Other NDDs is low, but especially for ASD, with rates described for CMA and NGS of 10–40%^4,8^. The main genetic contributors to ASD are rare inherited and de novo CNVs and SNVs^20–24^, being causality of de novo variants easier to determine. With the classification criteria followed in this study, based on the ACMG guidelines, novel or rare inherited variants, not associated before with ASD, remained as VUS. These variants accounted for 10.5% of patients with ASD by aCGH and 18.4% of patients by NGS. Also, 1.6% of ASD patients carried what we denominated a VUC (variants with incomplete penetrance and sex chromosomes aneuploidy) for which the exact contribution to the patients’ phenotype could not be accurately established. Criteria for considering a case carrying such variants solved or not was exclusively based on the performance of additional genetic tests after the aCGH, which indicates whether a clinical geneticist found the variant likely explaining the phenotype of the patient or not. This criterion might have led to inaccuracy in the diagnostic rate calculation as these variants could be contributing to the phenotypes but not taken into account.

The classification of patients in the diagnostic categories might have also affected the low rates found for ASD. This classification was made according to the patients’ clinical records, where, often, patients with ASD features are diagnosed as GDD due to either not being old enough to be diagnosed as ASD or not being assessed with a specific ASD-standardized scale to provide an accurate diagnosis. These patients might have been wrongly assigned as GDD (GDD/ID), a category with a greater diagnostic rate.

It has to be highlighted that this limitation is intrinsic to all NDDs, where symptom overlap between different entities is significant, as well as the heterogeneity of their clinical characteristics, causes, treatment responses, and outcomes; what makes it complex to distinguish one from another^1^.

For aCGH we found a substantially lower diagnostic rate, overall (5.6%) and per phenotype category, than the 15–20% previously reported^10–12,25,26^. These differences might be attributed to multiple factors such as the criteria used for patient selection, being our cohort characterized by a high clinical heterogeneity where the genetic contribution to the phenotype is expected to vary widely; the sample size studied; the timing of the study and therefore, the knowledge about the CNVs; and the variant classification criteria used which has evolved over time with the increasing knowledge about population genomics; and the CMA methods used in other studies and their resolution. In this study, we used a 60 K aCGH, the smallest of all commercially available arrays, what might have resulted in the lower diagnostic yield observed. Arrays with higher probe densities generally lead to an increase in the detection yield that is often accompanied by an associated increase in the number of VUS that are detected^26^. However, as most arrays used clinically, the 60 K aCGH has probes concentrated in clinically relevant genes, covering over 245 recognized genetic syndromes and over 980 gene regions of functional significance in human development, which allows for detection of smaller CNVs within disease-associated regions while minimizing the number of VUS. Nevertheless, comparison of the diagnostic yield of NGS with higher resolution arrays (i.e. 180 K, 400 K, or 1 M) may be necessary to confirm our results.

As with NGS, performance of aCGH improved when patients were grouped into the phenotype subcategories. Our classification criteria of GDD/ID, ASD, and Other NDDs into the different subcategories (isolated, epilepsy, micro or macrocephaly, and syndromic) was based upon the most frequently co-occurring clinical features, signs, and symptoms seen in our clinical practice. In general, diagnostic rates were higher in non-isolated forms of GDD/ID and ASD. This observation is in line with previous reports that associate dysmorphisms and/or congenital malformations combined with other clinical signs to an increased aCGH diagnostic rate^27^. Indeed, several authors have evidenced the predictor effect of congenital malformations, facial dysmorphic features, cephalic perimeter, and others, in finding a pathogenic variant by aCGH^27–31^. Taken all together, these data support the different contribution and influence of genetic factors in the various categories and subcategories of NDDs and evidence the need of stratifying patients when designing and applying algorithms for the genetic diagnosis of these conditions.

The use of a targeted gene panel, instead of WES or WGS, might have led to an underestimation of the diagnostic yield of NGS in our sample. The clinical exome sequencing panels used in this study, CES (Sophia Genetics) and TSO (Illumina), target 4490 and 4813 known disease-associated genes, respectively, versus the 22,000 genes of WES/WGS. When only considering NDD-specific genes, these two panels contain ~1400 of the over 2000 genes published by the Deciphering Developmental Disorders consortium (data accessed on June, 2020), which can lead to an underestimation of the diagnostic rate. Another disadvantage of the use of a targeted gene panel instead of WES/WGS is the fixed content of the former, not allowing the possibility of adding newly discovered genes or periodic reanalysis of the data with new evidence. This static content is a limitation, since NGS reanalysis of negative cases as new evidence is published has been described to increase 10–25% the diagnostic yield of this method^32–35^.

On the other hand, besides the easier interpretation of results and the minimal risk of incidental findings, a strength of using a targeted panel is the higher coverage of the genomic regions included, which allows for a more accurate screening of CNVs compared with WES. Indeed, all critical genes of microdeletion and duplication syndromes and most contiguous gene deletion syndromes found by aCGH in this study are included in the clinical exome sequencing panels used and therefore, potentially identifiable. Although the number of samples tested was limited (*n* = 29), our clinical exome sequencing method was able to detect all clinically relevant variants (*n* = 6), i.e. the pathogenic CNVs (*n* = 1), variants with incomplete penetrance (*n* = 3) and sex chromosomes aneuploidies (*n* = 2). CNVs not detected by the clinical exome panel were all VUS and the main cause for the not detection was the region not being targeted by the clinical exome sequencing panel, which is designed to include genes known to cause human disease. When only CNVs falling in regions captured by both methods were considered, the clinical exome sequencing CNV analysis was able to detect 90% of the variants.

The present study has several limitations, some of which have already been acknowledged, such as the possible overestimation of the clinical exome sequencing diagnostic yield due to a bias in the selection of samples for NGS and a lower than previously reported aCGH diagnostic yield. Another limitation of the study is its retrospective design. Although, our results are consistent with all previously published works on the diagnostic performance of NGS^5,7,13–16^, a prospective study comparing the diagnostic yield of aCGH and clinical exome sequencing in an unbiased sample would be desirable to confirm our results. Lastly, we did not evaluate factors related to the NGS implementation process such as cost, turn-around-time, acceptability by clinicians; ease of use; validation of results; or storage and management of genetic variants, which still pose some challenge for implementing diagnostic NGS^36^. However, clinical exome sequencing was implemented in our laboratory in 2016 and, although it was not specifically set-up for NDDs, such factors were assessed prior implementing NGS for the diagnosis of genetic diseases in our clinical practice.

Finally, it is worth mentioning that we did not find any positive result for Fragile-X syndrome across the 250 patients tested. Although the number of patients screened in this study is limited, recent evidence supports that the frequency of Fragile-X syndrome has been overestimated over time and now is calculated as 1% of all ID, with the majority of patients having either compatible clinical features or a family history suggestive of this disorder^37^

Considering the above-mentioned limitations, our results suggest that clinical exome sequencing could be useful in the genetic diagnosis of NDDs as a first-tier test in the diagnostic algorithm, especially nowadays when CNV analysis from NGS data is possible and methods are being increasingly optimized. NGS may be followed by aCGH in cases not solved or cases where the genomic region of a large CNV identified by NGS needs to be accurately delimited, and only perform Fragile-X testing in highly suspected cases.

## Methods

### Patients and samples

The project was approved by the ethics committee of FJD and was performed in accordance and the Declaration of Helsinki Principles and institutional requirements. Written informed consent was obtained from each participant or their guardians.

The selection of subjects was performed by retrospective review of patients with a diagnosis of NDD referred to our Genetics Department by the pediatric neurologist and/or neurologist and having an aCGH study done between January,1st, 2015 and December, 31st, 2019.

Patient information was extracted from the patients’ electronic health records. Data collected was sex, age, patient’s diagnosis; pregnancy and perinatal/neonatal history; data on imaging and electrophysiological tests; family history; laboratory findings and genetic tests performed.

Clinical diagnosis of NDD was performed by a pediatric neurologist or neurologist, depending on the age of the patient. Patients were evaluated by a clinical geneticist, which included a detailed anamnesis, pedigree analysis, and physical examination.

Genomic DNA from all patients was extracted from peripheral blood samples using automated DNA extractors: BioRobot EZ1 (QIAGEN, Hilden, Germany) or MagNA Pure Compact system (Roche Applied Science, Penzberg, Germany). Parental samples were obtained, when possible, to determine the origin of the genetic variants identified in the probands.

### NDD diagnostic algorithm and genetic analyses

The algorithm used for the genetic diagnosis of NDD did not change over the 4-year period from 2015 to 2019 and included an aCGH for all cases with NDD, isolated or accompanied by other symptoms, and not characterized genetically; followed or preceded by Fragile-X testing in patients with negative or inconclusive results when there was clinical suspicion of this condition. Patients were evaluated by the clinical geneticist upon completion of each of the above-mentioned tests and clinical exome sequencing was ordered in patients with non-informative or inconclusive results. Factors influencing the ordering of clinical exome sequencing include having a confirmed NDD diagnosis upon post-test evaluation, which occurs in over 90% of the cases; the complexity of the phenotype; the likelihood of having a monogenic cause based on the patient’ and family history; the possibility of follow-up and the interest of the patient and family to continue the genetic study, as some patients do not come back to the clinic after the aCGH result. Another factor is the timing of the clinical exome sequencing ordering since this technology was incorporated to our algorithm by the end of 2016 and therefore there is a substantial number of patients that are still awaiting to be subjected to exome sequencing.

### aCGH

aCGH was performed using the aCGX 60 K platform (CGX^TM^, PerkinElmer, Inc) following the manufacturer’s protocol. The platform has a resolution of 190 Kb in the backbone and 28 Kb in the targeted regions and covers over 245 recognized genetic syndromes and over 980 gene regions of functional significance in human development.

Quality control included several parameters such as DLR spread, reproducibility, background/signal intensity, and signal/noise ratio.

The array images were scanned and extracted using the SureScan Microarray Scanner (Agilent Technologies, Santa Clara, California, USA). CNV analysis was conducted with the Genoglyphix^®^ platform (PerkinElmer, Inc).

### Clinical exome sequencing and variant analysis

Since the implementation of clinical exome sequencing, two different technologies/capture designs have been used: the Illumina True Sight One (TSO; Illumina, San Diego, CA) at the end of 2016, and the Clinical Exome Solution v1 and v2 by Sophia Genetics (CES; Sophia Genetics, Boston, MA) from 2018 and 2019 respectively, to date. TSO and CES target 4813 and 4490 genes involved in human diseases, respectively, and have an overlap of 3815 genes. Both libraries were run on a NextSeq500 instrument (Illumina, San Diego, CA).

For sequencing data analysis of the TSO clinical exome, we used a standard pipeline that was run on the Illumina BaseSpace Sequence Hub and analyzed using the Variant Study platform (Illumina, San Diego, CA). For the CES, the NGS data analysis was performed using algorithms developed by Sophia Genetics and implemented in the SOPHiA DDM™ analysis platform and included both SNV and CNV identification. CNVs identified by aCGH in samples also sequenced with the CES clinical exome were specifically queried and CNV detection yield in these samples was calculated.

Overall quality control of the NGS data included the capture on the “on-target” region ≥60%, the coverage at ×25 ≥ 95% and ×50 ≥ 90%. Variant-level quality control included the flagging “PASS”, a read depth ≥20, and a frequency of the alternative allele ≥30%.

Variant analysis in all patients was performed using a virtual gene panel specific for NDD based on the list of genes regularly published by the Deciphering Developmental Disorders consortium (www.ddduk.org) and evidence gathered from the scientific literature. Both techniques were applied to index cases.

Variant filtering and prioritization was based on the following: the genotype of the variant and the suspected mode of inheritance; the minor allele frequency in the Genome Aggregation Database (gnomAD) ≤1% for recessive and X-linked and ≤0.5% for dominant genes; the functional effect of the variant in the protein (loss-of-function, missense, in-frame indels, and splicing variants); for missense variants, the predicted effect in silico; the description of the variant in ClinVar or the scientific literature; the existence of functional studies supporting the pathogenicity of a variant; and the results from segregation analyses.

### Additional genetic tests

Additional genetic tests were performed on some patients based on the clinical suspicion or the aCGH/NGS results found. These tests included the karyotype, Fragile-X screening by triplet-primed PCR; X-chromosome inactivation (XCI) testing the HUMARA locus^38^, Sanger sequencing for the familial segregation of variants identified by NGS; and MS-MLPA assay for confirmation of Prader–Willi and Angelman syndromes (ME028 SALSA Probemix, MRC Holland).

### Patients’ phenotype classification

For analysis purposes, patients were classified into different categories based on the clinical diagnosis made by the neuropediatrician and clinical geneticist at the time of referral and the specific area of neurodevelopment or central nervous system affected:

(1) GDD/ID: global developmental delay (age < 6 years) or intellectual disability (age ≥ 6 years); (2) ASD: autism spectrum disorder; (3) other neurodevelopmental phenotypes (Other NDDs) such as attention deficit hyperactivity disorder (ADHD), communication disorders; specific language disorder; specific learning delay; or primary motor delay.

The three categories were further subdivided into four subcategories, according to the most frequently co-occurring clinical features, co-morbidities, signs, and symptoms: (A) Isolated forms, if no other system was involved or forms with less than five dysmorphic features; (B) Forms with epilepsy: if only epilepsy or seizures were also present (excluding epileptic encephalopathy); (C) Forms with microcephaly or macrocephaly: if the only accompanying symptom was a cephalic perimeter below or above two standard deviations (SD), respectively; and (D) Dysmorphic/syndromic forms for patients with dysmorphic facial and/or skeletal features (at least five specific features) or patients with at least two or more of the following: epilepsy, micro/macrocephaly, stature and/or weight above or below 2 SD; cardiac, hearing, hormonal, neurological, skeletal, urogenital and/or vision anomalies. The number of five dysmorphic features chosen to stratify patients into syndromic or not syndromic was arbitrary but aimed to be high enough to assure a syndromic form and minimize the subjectivity of the observers. The different phenotype categories and subcategories are illustrated in Table 1.

### Variant interpretation and classification

Both aCGH and NGS variant interpretation was done at the time of testing and no new analysis was made specifically for this study.

Criteria for CNV classification were based on previous recommendations^39,40^ and included: (1) Type of CNV: deletion or duplication; (2) Size; (3) Region involved; (4) Gene density and function of the genes involved; (5) Frequency of the variant in the Database of Genomic Variants; (6) Origin of the variant (inherited versus de novo). (7) Partial or complete overlap with a known syndrome or well-established neurodevelopmental disease genes; (8) Previous classification of the CNV/gene in the databases and literature. Databases used for CNV classification and interpretation were the following: Database of Genomic Variants (DGV), DECIPHER, Human Genetic Mutation Database, Orphanet, the Simons Foundation Autism Research Initiative (SFARI) and NCBI databases (ClinGen Genome Dosage Map, ClinVar, International Standards for Cytogenomic Arrays -ISCA-, Online Mendelian Inheritance in Man –OMIM-, PubMed.

CNVs were classified into seven different classes (Fig. 1):

(A) Definitely pathogenic/causal CNVs: CNVs overlapping a specific syndrome or involving a well-established gene; CNVs previously reported as pathogenic or likely pathogenic in databases; unbalanced product of parental translocations; or other chromosomal rearrangements. (B) Likely pathogenic CNVs: Deletions and duplications not previously reported or reported in a limited number of cases involving genes previously associated with NDD, epileptic encephalopathy or brain congenital malformations. (C) Known pathogenic CNVs with incomplete penetrance: Deletions and duplications previously reported or reported in association with NDDs with a penetrance <100%. (D) Sex chromosome aneuploidies: Turner syndrome (monosomy X); triple X syndrome; Klinefelter syndrome (XXY); and XYY syndrome. (E) Variants of unknown significance (VUS): CNVs containing genes for which evidence of pathogenicity is not currently available or the exact contribution is not determined. (F) Unrelated finding: Pathogenic CNV in a recessive gene not related with the clinical indication of the study. (G) Benign/Likely Benign CNV: CNVs without genes or with genes previously described as polymorphisms.

Classes A to F were reported back to patients at the initial referral with the appropriate genetic counseling by a clinical geneticist. For analysis purposes, we only considered classes A to E CNVs.

SNVs resulting from clinical exome sequencing analysis were classified into the five different categories proposed by the ACMG/ESHG (Fig. 1): (1) Benign; (2) Likely Benign; (3) Variant of unknown significance (VUS); (4) Likely pathogenic and (5) Pathogenic^41,42^. Variant classes 3–5 were reported back to patients at the initial referral with the appropriate genetic counseling.

### Patient classification

Based on CNV and SNV classifications, patients were divided into four categories (Fig. 1): (i) Solved patients: patients carrying a pathogenic, likely pathogenic CNV (classes A and B) or SNV (classes 4 and 5) related to the referral reason or a variant of incomplete penetrance (CNV class C) explaining the phenotype of the patient. (ii) Patients with VUC: patients where the exact contribution of the variant is not clear. These patients were cases with a known pathogenic variant with incomplete penetrance (CNVs class C) where the phenotype associated with the variant did not fully explain the phenotype of the patient and additional genetic testing was ordered; cases with the 15q11BP1-BP2 deletion, with a well-established mild effect^43^; cases with sex chromosomes aneuploidy (CNVs class D); and females with X-linked pathogenic variants inherited from an unaffected mother. (iii) Patients with inconclusive results: patients with a VUS (CNVs class E and SNV class 3). (iv) Unsolved patients with a non-informative result: patients with no CNV identified by aCGH, an unrelated finding identified by aCGH (CNVs class F), or a likely benign or benign variant (CNVs class G and SNV classes 1–2).

Patients with more than one CNV or SNV identified were classified based upon the most pathogenic finding.

### Statistical data analysis

Descriptive statistics (mean and SD) were calculated for age at diagnosis. Diagnostic yield for aCGH and clinical exome sequencing was calculated as the number of solved patients per category and subcategory and expressed as percentage of the total number of patients in the corresponding category and subcategory. Comparison between the diagnostic yield of aCGH and clinical exome sequencing was performed using a logistic regression model with mixed effects to take into account the non-independence of the samples, and *p* values were corrected using the Benjamini–Hochberg method. The association of gender with the diagnostic yield and the comparison between the two exome sequencing tests used (TSO and CES) were performed using a Fisher exact test, for each phenotype category and subcategory. The statistical significance was set as *p* < 0.05.

### Reporting summary

Further information on experimental design is available in the Nature Research Reporting Summary linked to this paper.

## Supplementary information

Supplementary Information

Reporting Summary

## Data Availability

The sequencing data that support the findings of this study are available in the European Genome-Phenome Archive (https://ega-archive.org/): https://www.ebi.ac.uk/ega/studies/EGAS00001004949.

## Code availability

aCGH analysis was conducted with the Genoglyphix^®^ platform (PerkinElmer, Inc). Data analysis of the TSO clinical exome was run on the Illumina BaseSpace Sequence Hub and analyzed using the Variant Study platform (Illumina, San Diego, CA). For the CES, the NGS data analysis was performed using the SOPHiA DDM™ analysis platform.
