# Supplementary material for: Comparison of the diagnostic yield of aCGH and genome-wide sequencing across different neurodevelopmental disorders
Source: NPJ Genom Med. 2021 Mar 25;6:25. doi: 10.1038/s41525-021-00188-7 (PMC7994713; doi:10.1038/s41525-021-00188-7)
Supplement: Supplementary file 1 — Supplementary Information [file 41525_2021_188_MOESM1_ESM.pdf]

Supplementary Table 1. CNVs identified by aCGH in samples subjected to clinical exome sequencing and the detection or not by the CNV analysis

ID	CNV classification	Locus (gene)	Type of CNV	Genomic coordinates (aCGH)	CNV detected by clinical exome sequencing	Estimated genomic coordinates from clinical exome sequencing analysis	Comment
RM-0515	Pathogenic (Xq28 deletion)	Xq28	Deletion	chrX:149247622-155221913	Yes	chrX:(148586678_149613771)-(155240084_end)	--
RM-1194	Pathogenic-incomplete penetrance (15q13.3 microduplication)	15q13.3	Duplication	chr15:32029550-32438943	Yes	chr15:(31453196_32322788)-(32404110_33022881	--
RM-0066	Pathogenic-incomplete penetrance (NRXN1 deletion syndrome)	2p16.3 (NRXN1)	Deletion	chr2:50557535-50766750	Yes	chr2:50573823-50765779	--
RM-1682	Sex chromosome aneuploidy (XYY)	--	Duplication	--	Yes	chrX:(1_591623)–(2692828_2700078); chrX:(154774948_155119120)-(155235154_155239470)	--
V-2638	Sex chromosome aneuploidy (XYY)	--	Duplication	--	Yes	chrX:(1_591623)- (2692828_2712550); chrX:(154774948_155119120)-(155235863_155237170)	--
RM-1275	15q11.2 BP1-BP2 microdeletion	15q11.2 BP1-BP2	Deletion	chr15:22822019-23085219	Yes	chr15:(1_22833515)-(23086383_23889129	--
RM-0839	VUS	q28	Duplication	chrX:148880773-149367334	No	--	Region not targeted by CES
RM-0839	VUS	10q23.2	Duplication	chr10:88587545-88705602	Yes	chr10:(88492749_88635750)-(88683486_89264662	--
XF-0891	VUS	16p13.11	Deletion	chr16:15053284-15154175	Yes	chr16:(14766617_15219538)-(15231790_15489970)	--
RM-1416	VUS	16p13.3 (*RBFOX1*)	Duplication	chr16:6873755-6935092	No	--	Intronic variant (not targeted by CES)
RM-0572	VUS	16p13.3 (*RBFOX1*)	Deletion	chr16:6893768-6953471	No	--	Intronic variant (not targeted by CES)
RM-1491	VUS	16q23.3	Duplication	chr16:82524260-83138488	No	--	Gene in target region (CDH13) covered by CES but not detected by the CNV analysis algorithm
RM-1461	VUS	17p11.2	Deletion	chr17:19142083-20143948	Yes	chr17:(18259308_19240967)-(20217393_20397286)	--
RM-0694	VUS	17p13.3	Deletion	chr17:1326321-1487047	Yes	chr17:(1257652_1371110)-(1465893_1554085	--

RM-0132	VUS	17q12	Duplication	chr17:31962547-32937481	Yes	chr17:(30815573_32582347)-(32614719_33310014)	--
RM-0622	VUS	1q42.2 (*DISC1*)	Duplication	chr1:231734070-231806283	No	--	Intronic variant (not targeted by CES)
RM-0918	VUS	1q42.2 (*DISC1*)	Duplication	chr1:231719623-231806283	No	--	Intronic variant (not targeted by CES)
V-1809	VUS	22q11.21	Deletion	chr22:20033538-20060533	No	--	Region not targeted by CES
XF-1046_1	VUS	2p16.3 (*NRXN1*)	Deletion	chr2:50986477-51096409	No	--	Intronic variant (not targeted by CES)
XF-1046_2	VUS	2p16.3 (*NRXN1*)	Deletion	chr2:50986477-51096409	No	--	Intronic variant (not targeted by CES)
RM-0432	VUS	2q21.1	Deletion	chr2:131483022-131886567	No	--	Region not targeted by CES
V-2711	VUS	2q24.1	Deletion	chr2:156730580-157218918	Yes	chr2:(155307074_157182246)-(157186708_157332608	--
RM-1335	VUS	3p25.2 (*RAF1*)	Duplication	chr3:12630854-12799925	Yes	chr3:(12627314_12632282)-(12660235_13860430)	--
RM-0839	VUS	3p25.2 (*RAF1*)	Duplication	chr3:12630854-12799926	Yes	chr3:(12627314_12632282)-(12660235_13860430)	--
RM-1423	VUS	4q24	Duplication	chr4:103898527-104624966	Yes	chr4:(103806598_104510829)–(104640842_106155089)	--
RM-1069	VUS	4q28.3	Deletion	chr4:136421277-138398102	No	--	Region not targeted by CES
RM-1615	VUS	7q35	Deletion	chr7:146213130-146293204	No	--	Intronic variant (not targeted by CES)
RM-1460	VUS	9p24.3	Duplication	chr9:209020-327369	Yes	chr9:(1_271612)–(340336_370205)	--
RM-0506	VUS	9q21.33-q22.1	Duplication	chr9:88778748-91703527	No	--	Only one gene from the target region included in CES (*GAS1*) but not detected by the CNV analysis algorithm
RM-0365	VUS	Xp22.31	Deletion	chrX: 6,456,777-8,119,329	Yes	chrX:(6069518_6451774)–(7268312_8502349)	--
RM-1160	VUS	Xp22.33	Duplication	chrX:359396-600393	Yes	chrX:(1_591623)–(595570_601524)	--
